# The genetic architecture underlying prey-dependent performance in a microbial predator

**DOI:** 10.1038/s41467-021-27844-x

**Published:** 2022-01-14

**Authors:** Balint Stewart, Nicole Gruenheit, Amy Baldwin, Rex Chisholm, Daniel Rozen, Adrian Harwood, Jason B. Wolf, Christopher R. L. Thompson

**Affiliations:** 1 Centre for Life’s Origins and Evolution, Department of Genetics, Evolution and Environment, University College London, Darwin Building, Gower Street, London, WC1E 6BT UK; 2 Cardiff School of Biosciences, Neuroscience and Mental Health Research Institute (NMHRI), Cardiff University, Hadyn Ellis Building, Maindy Road, Cardiff, CF24 4HQ UK; 3 Feinberg School of Medicine, Northwestern University, Chicago, IL 60611 USA; 4 Institute of Biology, Leiden University, Sylvius Laboratory, Sylviusweg 72, PO Box 9505, 2300 RA Leiden, The Netherlands; 5 Milner Centre for Evolution and Department of Biology and Biochemistry, University of Bath, Claverton Down, Bath, BA2 7AY UK

**Keywords:** Evolutionary genetics, Mutagenesis, Evolutionary biology

## Abstract

Natural selection should favour generalist predators that outperform specialists across all prey types. Two genetic solutions could explain why intraspecific variation in predatory performance is, nonetheless, widespread: mutations beneficial on one prey type are costly on another (antagonistic pleiotropy), or mutational effects are prey-specific, which weakens selection, allowing variation to persist (relaxed selection). To understand the relative importance of these alternatives, we characterised natural variation in predatory performance in the microbial predator *Dictyostelium discoideum*. We found widespread nontransitive differences among strains in predatory success across different bacterial prey, which can facilitate stain coexistence in multi-prey environments. To understand the genetic basis, we developed methods for high throughput experimental evolution on different prey (REMI-seq). Most mutations (~77%) had prey-specific effects, with very few (~4%) showing antagonistic pleiotropy. This highlights the potential for prey-specific effects to dilute selection, which would inhibit the purging of variation and prevent the emergence of an optimal generalist predator.

## Abstract (web summary)

What prevents a generalist predator from evolving and outperforming specialist predators? By combing analyses of natural variation with experimental evolution, Stewart et al. suggest that predator variation persists because most mutations have prey-specific effects, which results in relaxed selection

## Introduction

The fitness of individuals within a predator species should be closely tied to their relative ability to convert prey resources into the production of progeny. Consequently, mutations that improve predatory performance would be expected to rapidly spread through a predator population, eroding genetic variation within species. However, intraspecific variation in predator traits is widespread^1^. A key problem is therefore to identify the processes that allow genetic variation to persist by preventing the emergence of a superior generalist predator that can outcompete others across all prey types^2–6^.

Diversity within predator populations can be maintained if different prey types or species impose different selective pressures, such that different variants of a predator species could evolve to specialise on different prey. The extent to which specialisation will evolve within predator populations ultimately depends on the interplay of genetic and environmental factors. For example, if mutations generally increase fitness in one environment but decrease fitness in another environment (antagonistic pleiotropy), then exposure to fluctuating environments would result in fitness trade-offs^7^, which would be manifested as negative genetic correlations in performance across different ecological conditions. However, field studies across a range of organisms rarely show strong evidence for such trade-offs and performance in one environment is typically poorly correlated with performance in another^8–12^. Similarly, a wide variety of experimental evolution studies in microbial systems have shown that de novo adaptive mutations in ‘home’ environments are generally beneficial or neutral (rather than antagonistic) in alternative ‘away’ environments^13–19^. Consequently, there is increasing interest in alternative processes that can allow for the maintenance of trait variation. One compelling possibility is that mutations only have effects in some environments and therefore are only under selection when that environment is encountered. For example, mutations that have deleterious effects on predation on one prey type could be neutral on other prey types (conditional neutrality)^20^. Conditional neutrality can promote the persistence of variation and ecological specialisation because it results in the overall relaxed selection, which limits the degree to which selection purges deleterious mutations^21–25^. The importance of this process will depend on the extent to which the availability of different prey types exhibits spatial and/or temporal variability, as this will determine the extent to which the influence of selection arising from each prey type is diluted.

The relative impact of antagonistic pleiotropy and/or relaxed selection on genetic diversity and ecological specialisation in natural systems, such as predator populations, is still poorly understood. It is often difficult to obtain detailed information about the spatial ‘graininess’ or temporal variability of the environment (how often alternative prey is experienced)^2,4^. There are also considerable technical challenges to generating quantitative biologically relevant measures of predatory performance and to link them to fitness variation for the same individuals^26^. Finally, studies of predator–prey interactions often focus on predators that are complex non-model species, making it difficult to link any measures of performance and fitness variation to genetic variation, leaving us with a limited empirical understanding of the evolutionary genetics of predatory performance^27,28^. Consequently, it is generally unknown how adaptation to one prey type impacts fitness on other prey types, and whether new mutations affecting predation are generally pleiotropic or conditionally neutral. Model microbial predators potentially provide powerful systems for studying the evolutionary processes that can maintain genetic variation in predatory performance in populations^29–31^. The relative abundance of different genotypes when consuming different prey types can be tracked through generations to estimate their relative performance as predators across the different prey. Moreover, they are genetically and molecularly tractable, providing direct links between genetic differences and realised lifetime fitness variation. The social amoeba *Dictyostelium discoideum* offers a particularly powerful and compelling model microbial system. *D. discoideum* is an efficient predator of diverse bacterial prey in soil ecosystems, where local competition for bacterial prey among different genotypes is likely to be an important source of persistent selection^32^. Different *D. discoideum* genotypes are known to coexist at a fine-scale^33^ and there is an elaborate mechanism where amoebae aggregate to form a fruiting body that facilitates dispersal (via spores) away from environments in which prey have become depleted, indicating that prey is often a limiting resource. Because spore dispersal appears to be efficient^34^, genotypes are also likely to experience a broad array of prey types, providing ample opportunities for selection across different prey to shape predatory traits. Indeed, a recent metagenomic survey has illustrated the tremendous diversity of potential bacterial prey that *D. discoideum* associates within their natural environments^35^. Finally, successful predation by *D. discoideum* requires the integration of numerous complex biological processes, such as those that allow amoebae to detect, move towards, engulf and digest a range of bacterial prey, as well as mechanisms to overcome prey evasion and virulence^36^. Different prey types could, therefore, generate very different patterns of selection on predatory traits in *D. discoideum*, which has the potential to promote specialisation on different prey types and maintain genetic diversity. However, the realised evolutionary outcome will depend on the interaction between the pattern of prey-induced selection and the genetic architecture of predation related traits, which together govern how selection acts on mutational variation.

To achieve a broad understanding of the pleiotropic properties of genetic variation associated with predation across different contexts we have integrated complementary approaches using naturally occurring variation and experimentally induced mutational variation in *D. discoideum*. Quantification of predatory success of *D. discoideum* natural isolates known to co-occur in nature on a representative range of bacterial prey that is naturally encountered allowed us to understand the properties of standing variation in nature. We found that natural strains show widespread reversals in their relative predatory success across different prey (i.e. they frequently change in their rank order of fitness on different prey). To understand the impact of newly arisen mutations (which have not already been screened by natural selection) on predatory success across different bacterial prey, we developed an experimental evolution approach (REMI-seq)^37^. REMI-seq allows next-generation sequencing to be used to simultaneously quantify the fitness effects of thousands of mutations on predation performance on different prey species. These laboratory-generated mutations largely result in prey-specific effects, with only a small minority showing antagonistic pleiotropy. Our results support the idea that environmental heterogeneity and the conditional nature of the effects of mutations result in relatively weak selection on variation affecting performance on each prey. These results are consistent with the idea that relaxed selection, rather than antagonistic pleiotropy associated with trade-offs, better explains how genetic variation in predatory traits is maintained, despite the potential for recurrent selection favouring superior predators.

## Results

### Natural *D. discoideum* strains vary in growth rate on a single bacterial prey

**Fig. 1:**
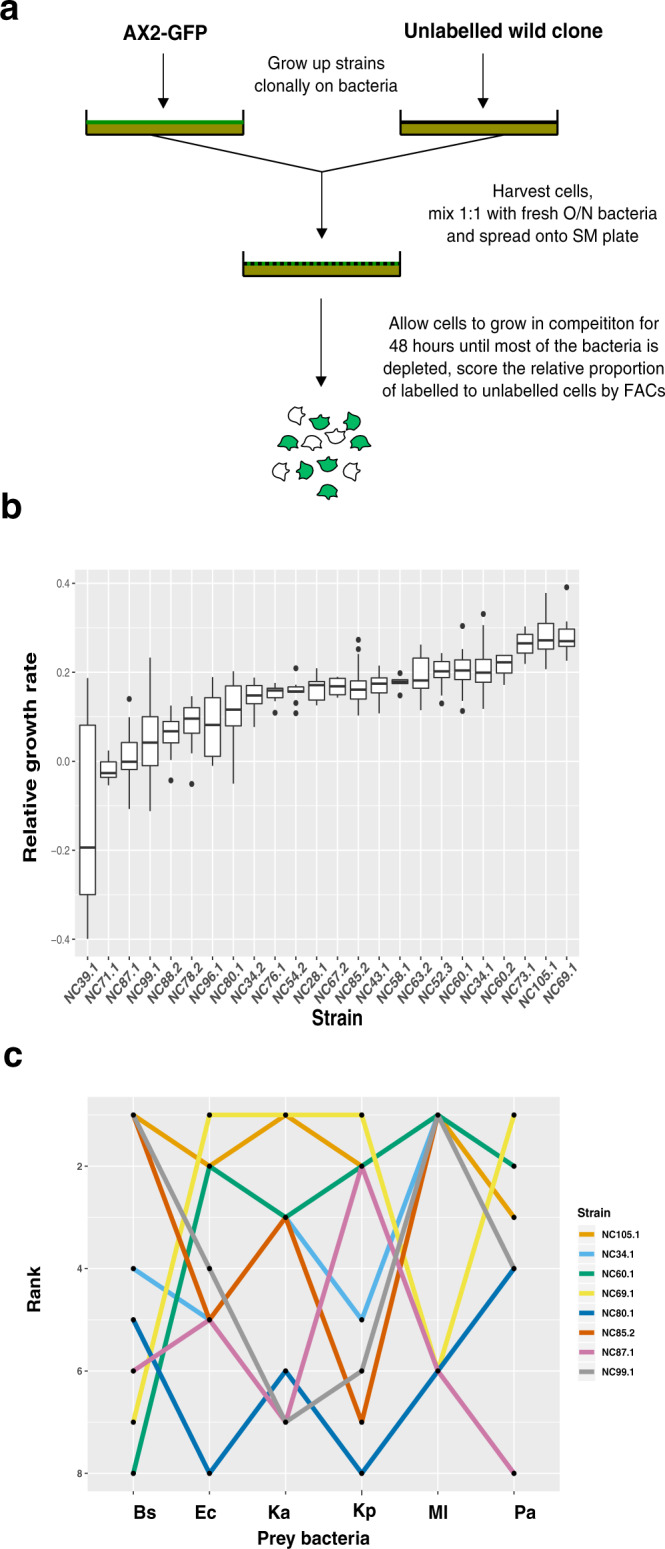
Analysis of *D. discoideum* natural isolate growth on different bacterial prey. **a** Schematic of growth competition assay of natural strains. Natural isolates and the reference strain AX2-GFP were initially grown up clonally. Each natural isolate was then mixed with AX2-GFP at a starting ratio of 1:1 and plated onto an SM plate with bacterial prey as a food source. The starting frequencies of the two strains were determined by flow cytometry. Mixtures of strains were grown in competition for 48 h (~10 generations of growth) and the relative proportion of labelled and unlabelled cells was determined by flow cytometry. **b** Growth of *D. discoideum* natural isolates compared to AX2-GFP on *K. aerogenes*. Growth rates are relative to the AX2-GFP control. Horizontal lines within the boxes indicate median values, the boxes the interquartile range, whiskers show the smallest and largest values within 1.5 times the interquartile ranges above and below the 75th and 25th percentile, and dots represent outliers from these ranges. Competitions were performed between 4 and 23 times for each of the 24 wild *D. discoideum* strains (median *n* = 11). **c** Frequent switches in competitive performance occur across different prey types. Relative growth rates for a representative subset of eight natural isolates were determined on six bacterial prey: *K. aerogenes* (Ka), *B. subtilis* (Bs), *E. coli* (Ec), *K. pnuemoniae* (Kp), *M. luteus* (Ml), and *P. aeruginosa* (Pa), and then ranked (1 = best performer, 8 = worst) based on their “win-loss” record (in terms of significant differences in growth) when compared to each other strain on a given bacteria (see ‘Methods’ section). Each line shows the ranking of one of the eight different *D. discoideum* strains across all six bacterial prey types, with lines frequently crossing one another illustrating the frequent significant switches in performance ranking on different bacterial prey. At least three independent biological replicates were performed for each *D. discoideum* strain/bacterial prey combination competition. Source data are provided in the Source Data file.

To understand whether differences in predatory performance in *D. discoideum* might affect population dynamics, we first compared their relative growth rates on a single bacterial prey. The 24 natural strains we tested were all co-isolated from a single small geographic location in North Carolina^33^ and so are representative of the local co-existing biodiversity. *K. aerogenes* was chosen as the prey because it is commonly found in association with *D. discoideum* isolated from different environments^35^ and is widely used for routine *D. discoideum* culture. The growth of each strain over 48 h (about 10 generations) was measured in competition against a standard GFP-labelled laboratory strain (AX2-GFP), which was derived from the same location as our natural strains (Fig. 1a). The use of a standard competitor allowed for more sensitive detection of small differences in growth rate and the mitigation of block effects. We found that the 24 different strains show significant variation in their relative growth rates on *K. aerogenes* (one-way ANOVA F_23_ = 20, *p* < 2×10^−16^) (Fig. 1b), with >2.5 fold difference in relative growth between the strains showing the highest (NC69.1) and lowest (NC39.1) growth rates. To ensure that these differences accurately reflect variation in predatory efficiency of the strains (rather than interference competition with the standard competitor), the clonal growth rates of six different genotypes (representing examples from the top, middle and bottom of the growth hierarchy) were measured. Cells of each strain were plated clonally with *K. aerogenes* and the total number of cells counted following 48 h of growth (Supplementary Fig. 1A). Estimates of clonal growth are significantly correlated with competitive performance against AX2-GFP (*r* = 0.98, *p* < 0.001, Supplementary Fig. 1B), which supports the conclusion that *D. discoideum* strains exhibit differences in vegetative growth rate, and that these are driven by resource competition rather than interference competition.

### Swaps in predatory performance of *D. discoideum* strains on alternative prey

*D. discoideum* amoebae encounter diverse bacterial prey in nature^38^. We, therefore, tested whether growth on *K. aerogenes* reflects growth on other prey, or whether differences in predatory success across different prey species could potentially facilitate the maintenance of variation. The relative growth rates of eight representative *D. discoideum* strains from the analysis of growth on *K. aerogenes* were measured on a panel of six different bacterial species from diverse phyla, including proteobacteria, actinobacteria and firmicutes. These species were chosen to capture a representative spectrum of the different types of bacteria that have been co-isolated with *D. discoideum* and can be used as a growth resource^35,39^. This set includes four Gram-negative [Gram(−)] species (*K. aerogenes, Klebsiella pneumoniae, Escherichia coli* and *Pseudomonas aeruginosa*) and two Gram-positive [Gram(+)] bacterial prey species (*Bacillus subtilis* and *Micrococcus luteus*). This panel allows us to capture variation in predator performance associated with both highly divergent species as well as very closely related congeners. Together, this panel allows us to capture biologically meaningful variability in predator performance across prey with potentially different properties, as well as those with potentially very similar properties, to gain insights into the scale of variability.

We established a dominance hierarchy for growth on each prey species by ranking the strains of *D. discoideum* based on the number of strains that they show significantly higher versus lower relative growth, providing a sort of ‘win-loss’ count for each pair of strains (see ‘Methods’ section). Strikingly, we find frequent swaps in the dominance hierarchy across different bacterial prey (Fig. 1c and Supplementary Fig. 2). The pattern of changes in rank order is highly variable across prey, such that one strain does not perform significantly better than all others across every prey type, and similarly, no strain does significantly worse than all others over all prey types. Using the ‘win-loss’ record of each strain against each of the other strains, we find that, for 21 of the 28 strain pairs, there is a swap in their ranking of relative growth rate across at least one prey species (Source Data File). Even if we more conservatively only consider cases where a given pair of strains show a significant swap in their relative growth rate (meaning each strain shows both significantly higher and significantly lower growth than the other across at least two different species of bacterial prey), we find that 16 of the 28 pairs show significant reversals (Source Data File). Hence, in the majority of cases, each strain will both ‘lose’ to and ‘beat’ each other strain (in terms of growth rate) on at least one prey type.

Extensive performance swaps can arise if predatory performance is either strongly negatively correlated across some prey or if predatory performance is simply unpredictable and weakly correlated across prey. To distinguish between these possibilities, we calculated the rank correlations of strain performance across different prey (Table 1). We find that the correlations between performance rank on the different Gram(−) prey are all positive, with five of the six pairwise correlations being significantly different from zero (and the sixth, between performance rank on *K. aerogenes* and *K. pneumoniae*, showing a correlation of 0.62 with *p* = 0.1, suggesting it may also be positive). However, performance ranks on the Gram(−) are not correlated to performance ranks on the Gram(+), and the correlation between the performance rank on the Gram(+) is not significantly different from zero. These results are consistent with the notion that performance across Gram(−) bacteria reflects the presence of positive pleiotropy, while there is little evidence to suggest a link between performance on Gram(+) and Gram(−), nor between the alternative Gram(+) species. Despite the presence of positive correlations across performance on the Gram(−) bacteria, we still see widespread performance swaps across the different bacteria. A permutation test indicates that the number of performance swaps observed (21/28) is not significantly different to that expected under a hypothesis that performance across bacterial prey is random (observed reversals = 21, expected median reversals = 25, one-tailed *p* = 0.11). Thus, despite the fact that we have only sampled a relatively small set of potential bacterial prey, which likely only represents a limited range of prey characteristics, we nonetheless find a pattern that is consistent with the lack of a universally dominant competitor.

**Table 1:** Spearman’s rank correlation of average performance of natural strains across different bacteria.

	Ec	Ka	Kp	Pa	Bs	Ml
Ec		**0.71**	**0.79**	**0.88**	−0.24	0.12
Ka	0.0465		0.62	**0.90**	−0.24	0.05
Kp	0.0208	0.1017		**0.74**	−0.62	−0.10
Pa	0.0039	0.0020	0.0366		−0.36	0.14
Bs	0.5702	0.5702	0.1017	0.3851		0.19
Ml	0.7789	0.9108	0.8225	0.7358	0.6514	

Values above the diagonal are the rank correlations and the values below the diagonal are the corresponding *p*-values (unadjusted). Bacterial species are indicated by the first letter of the genus and species name [*K. aerogenes* (Ka)*, B. subtilis* (Bs), *E. coli* (Ec), *K. pnuemoniae* (Kp), *M. luteus* (Ml), and *P. aeruginosa* (Pa)] and are grouped by Gram status (Gram(−) = Ec, Ka, Kp, Pa; Gram(+) = Bs, Ml). Significant correlations are in bold. Source data are provided in the Source Data file.

### Fitness reversals across different prey can promote the persistence of genotypes in mixed environments

**Fig. 2:**
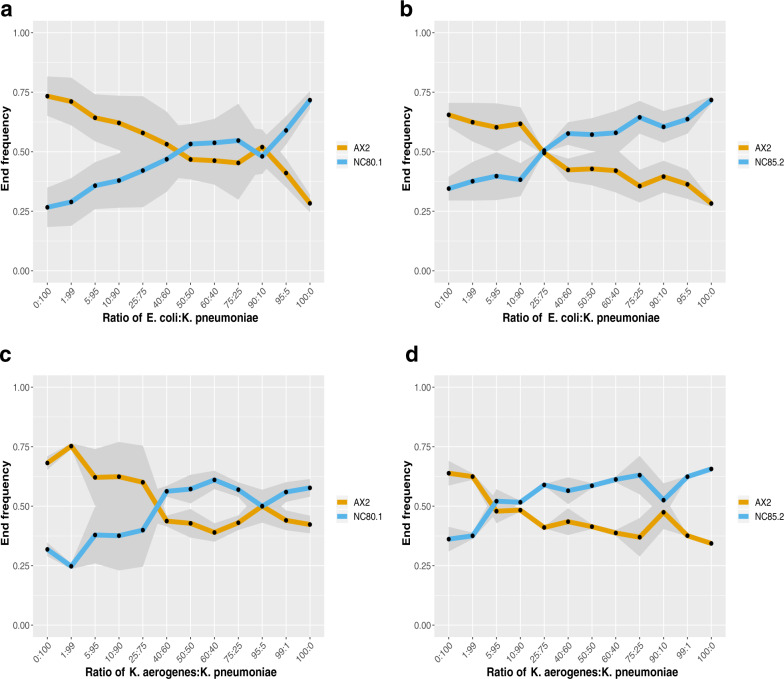
Strains can coexist after short-term growth on prey mixtures. Competitions were performed between NC80.1 (**a**, **c**) or NC85.2 (**b**, **d**) with AX2-GFP at a starting ratio of 1:1 on plates containing varying proportions of either *E. coli* with *K. pneumonia* (A & B) or *K. aerogenes* with *K. pneumonia* (**c**, **d**). Cells were allowed to grow together in competition for 48 h (~10 generations of growth). The relative frequency of each strain was quantified at the start and endpoints of the competition by flow cytometry. Individual data points (black circles) represent mean values and the shaded area is the standard error from two independent experimental blocks. Source data are provided in the Source Data file.

The fitness reversals we observed in the growth of different natural strains could facilitate the coexistence of different genotypes in nature, providing a mechanism for the persistence of genetic diversity. To further test this prediction, we studied the growth of strains of *D. discoideum* in the presence of a mixture of prey. Two natural isolates of *D. discoideum* (NC80.1 and NC85.2) were chosen that showed changes in rank order in growth across different prey against the AX2-GFP competitor strain. We examined patterns of coexistence for several generations (~10) across different mixtures (in terms of frequency) of prey (Fig. 2). In control experiments, in which a single bacterial food source is supplied, the strain with the higher measured relative growth rate outcompeted the other as expected (Fig. 2). However, when multiple prey species were available, there is a range of conditions (over a range of proportions of the different bacteria) that permitted the *D. discoideum* strains to coexist (Fig. 2). This experiment, carried out over ~10 generations of growth, provides further support for the prediction that reversals in competitive performance of foraging amoebae on different prey types could reduce fitness differences between the competitors, which could potentially stabilise persistence in environments with a diverse prey base over longer time scales.

### REMI-seq parallel phenotyping of thousands of insertion mutants on different prey

Natural isolates of *D. discoideum* exhibit widespread variation in predatory performance and we see frequent significant reversals in fitness across prey. This pattern suggests that the genes and biological processes required for growth (e.g. detection, capture, resistance to toxicity, digestion) are shared for some prey but not for others. In this case, patterns of predatory performance would reflect the weighted average pattern of pleiotropy (weighted by allele frequency and effect size). The positive correlations we see across the Gram(−) bacteria suggests the presence of positive pleiotropy, where mutations have the same direction of effect on different prey (whether it is beneficial or deleterious overall). However, the fact that we still see a large number of reversals in the hierarchy of growth even within the Gram(−) class suggests that there will not be universally positive pleiotropy. Such a pattern could be achieved either by a mixture of positively pleiotropic and some non-pleiotropic loci, or a mixture of positive and antagonistic pleiotropy (such that the weighted average is generally positive). Similarly, the lack of significant correlation between growth on any of the Gram(+) with the Gram(−) bacteria, and the lack of a significant correlation across the Gram(+) bacteria suggest that the average pattern of pleiotropy will be close to zero. Such a pattern could either reflect an overall lack of pleiotropy across these conditions, or else a balance of positive and antagonistic pleiotropic effects that effectively cancel one another out across prey. While these different patterns of pleiotropy can manifest as similar genetic correlations in nature, they have very different evolutionary explanations and consequences. If pleiotropy is generally low, mutations can be largely conditionally neutral, which can lead to relaxed selection at a locus that allows for the accumulation of mutational variation when growth occurs on prey where the locus has no effect^20^. Alternatively, if a locus shows antagonistic pleiotropy, then mutation frequencies can be pushed up and down across different prey depending on whether the variant is beneficial or deleterious on that prey (leading to a sort of balancing selection).

**Fig. 3:**
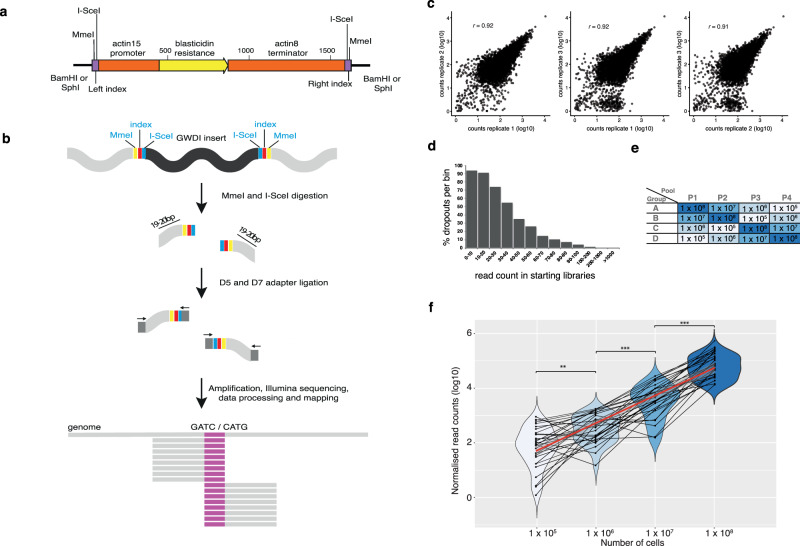
REMI-seq can be used to quantify the relative abundance of mutants. **a** Schematic of amplified insert for REMI mutagenesis. The insert was PCR amplified from the pGWDI plasmid^37^. **b** The REMI-seq method for insertion point identification. The GWDI fragment containing a blasticidin resistance cassette (black) inserts into the *D. discoideum* genome at DpnII or NlaIII loci. The termini of the GWDI insertion fragment contain recognition sequences for the type II restriction enzyme MmeI (yellow) and the meganuclease *I-Sce*I (blue). Digestion with *Mme*I and *I-Sce*I results in the generation of two 47–48 bp fragments. These fragments can be enriched by PCR and gel extraction, thus allowing identification of 19/20 bp of genomic sequence at the site of insertion by sequencing. **c** REMI-seq exhibits high technical reproducibility. Three preparations of gDNA from a pool of ~13,000 mutants were sequenced. Normalised read counts for each mutant are highly correlated across each replicate (*r* = 0.92; *r* = 0.92; *r* = 0.91, *p* < 2.2 ×10^−16^ for all comparisons). **d** Technical reproducibility and dropout rate is dependent on read count. Mutants were divided into bins dependent on the average starting read count between all replicates of the starting library. The percentage of mutants that could not be detected (dropped out) in one or more library was calculated for each bin. **e** Schematic of the experiment to determine the quantitative dynamic range of REMI-seq. Cells of 32 defined mutants were distributed into four groups (A, B, C, D) of eight mutants each (Supplementary Data 1). 10^5^, 10^6^, 10^7^, or 10^8^ mutant cells from each group were mixed to form four different pools (P1–P4). **f** REMI-seq can be used to quantify mutant abundance over >100 fold range. Pools of mutants spiked at different frequencies (P1–P4) were subjected to REMI-seq. Violin plots show the number of cells (log_10_ transformed) was highly correlated with normalised read counts (*r* = 0.55, *p* = 2.439 ×10^−11^). All means are significantly different to each other (one-sided *t*-test, 10^5^ vs 10^6^: *p* = 0.002; 10^6^ vs 10^7^: *p* = 0.0002; 10^5^ vs 10^6^: *p* = 0.002; 10^7^ vs 10^8^: *p* = 0.0002). See Supplementary Data 1 for separate results of each group of mutants.

Testing these ideas requires an understanding of the genetic architecture of predatory performance and the patterns of pleiotropy. This can be achieved by using experimental evolution to identify individual mutations that affect predatory performance and then compare their effects on different prey. However, since *D. discoideum* has one of the lowest mutation rates of all organisms measured to date^40^ and relatively large genome size (which results in a large mutational target size) it would be extremely difficult to identify a sufficient number of adaptive de novo mutations in experimentally evolved lineages to draw meaningful conclusions about the patterns of pleiotropy or the underlying pathways. Similarly, chemical mutagenesis approaches are limited by the number of mutations that can be identified and to phenotypes governed by a small number of genes^41^. REMI-seq^37^, (based on Tn-seq^42^) provides a means to overcome many of these limitations because it allows experimental evolution and mutation identification to be performed *en masse* on a pool of *D. discoideum* insertion mutants (Fig. 3a, b). Because REMI insertional mutants can generate null alleles they might be expected to differ in the magnitude of mutational effect and degree of pleiotropy compared to point mutations. However, hypomorphic alleles and gain-of-function alleles^43,44^ also occur, whilst recent studies in yeast suggest that genes and pathways identified by genome-wide analysis of deletion and amplification mutations extensively overlap with de novo adaptive mutations (such as SNPs) in experimentally evolved lines^45^. It is also important to consider that, even though beneficial loss-of-function mutants would be expected to show antagonistic effects in some context, there is no a priori reason to expect that fitness costs would be realised across different prey types. Furthermore, because this approach enables us to rapidly screen thousands of insertion mutants and captures a large fraction of the genes in the genome, we are able to test at a broad scale whether variants in individual genes tend to have shared or specific effects on predation across different prey. This provides a perspective on patterns of effects of mutations that have not been through the sieve of natural selection in the wild, providing an arguably less biased perspective on the nature of pleiotropic links between traits than does natural mutational variation. Finally, this broad-scale perspective also allows for a better understanding of the potential pathways underlying variation in prey utilisation, as well insight into the mechanisms underlying their pleiotropic consequences.

**Fig. 4:**
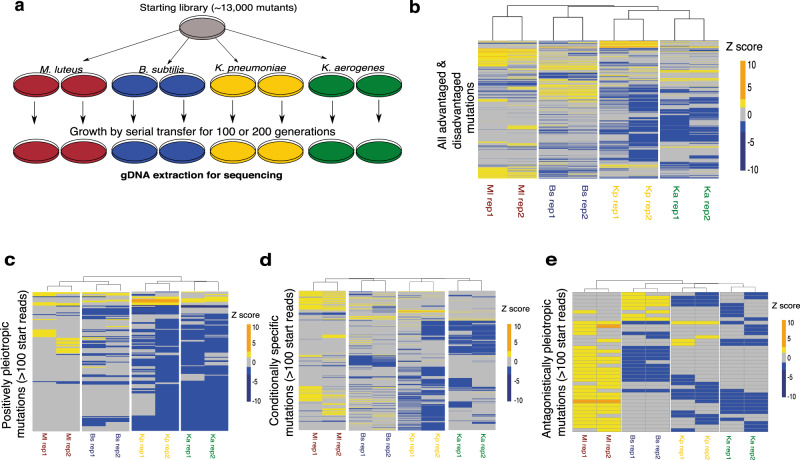
Parallel genetic selections reveal most mutations result in prey-specific effects. **a** Selection schematic. Cells from the mutant library were plated in duplicate in four parallel selections on the Gram(−) bacterial species *K. aerogenes* and *K. pneumoniae*, and the Gram(+) species *B. subtilis* and *M. luteus*. Cells were grown for either 100 generations (*B. subtilis*, *M. luteus*, *K. pneumonia* selections) or 200 generations (*K. aerogenes* selection) before being sequenced by REMI-seq to quantify the relative abundance of every mutant remaining in the pool (Supplementary Figs. 3,4 and Supplementary Data 2). **b** Patterns of behaviour across the different bacteria for mutants with a growth phenotype show an overall pattern of prey specificity. *z*-scores of mutants that were identified as either advantaged (yellow = mean *z* > 1.5, >100 normalised reads in both selection endpoint replicates) or disadvantaged (blue = mean *z* < −1, >100 normalised reads in the starting library) in one or more selections are shown across all four different bacteria (Ml = *M. luteus*, Bs = *B. subtilis*, Kp = *K. pneumoniae*, Ka = *K. aerogenes)*. **c** Mutations showing positive pleiotropy tend to be disadvantaged across multiple prey, and especially across the closely related prey species *K. aerogenes* and *K. pneumoniae*. The behaviour of mutants that were identified as positively pleiotropic (either advantaged on more than one prey or disadvantaged on more than one prey) with >100 normalised starting reads are shown. **d** Most mutations that affect growth show conditional specificity. The behaviour of mutants across all four bacterial prey showing conditional specificity (advantaged or disadvantaged on a single prey species only) with >100 normalised starting reads are shown. **e** Antagonistically pleiotropic mutations show little pattern. The behaviour of mutants that were identified as advantaged in a selection and disadvantaged in at least one other is shown across all four different bacteria. The majority of antagonistically pleiotropic mutants were advantaged in the *M. luteus* selection and disadvantaged on different prey.

After confirming the quantitative reproducibility of the REMI-seq technique (Fig. 3c–f and Supplementary Data 1), we performed parallel selections in duplicate across four different bacterial species: the Gram(−) species *K. aerogenes* and *K. pneumoniae*, and the Gram(+) species *B. subtilis* and *M. luteus* (Fig. 4a). These bacteria were chosen because they captured the majority of the fitness reversals in growth seen with natural *D. discoideum* strains, with 20 of the 28 strain pairs showing rank reversals across these four species of bacteria (compared to 21 of the 28 showing reversals across the complete set of bacterial species). Moreover, 15 of the strain pairs show significant swaps in their performance on this subset of bacteria (meaning each is both significantly higher and significantly lower than the other across at least one of these four bacteria; compared to 16 cases across the complete set of bacteria). We sequenced and counted reads for all mutants in the starting library and selection endpoints (after ~100 generations of growth for each selection, with the exception of the selection on *K. aerogenes* which was continued for ~200 generations). Mutants that have a selective advantage are expected to increase in frequency over the course of the selection relative to neutral mutations, whereas those with a disadvantage will decrease (see ‘Methods’ section). The starting library contains 12,479 unique single gene insertion mutants. In total, we identified 1170 mutations (representing 9.4% of all genes in the library) that significantly increased or decreased in frequency on one or more prey (Fig. 4b).

To confirm that the patterns inferred from the REMI-seq method provide quantitative information about the performance of mutants, we experimentally validated a set of mutants. Four mutants were chosen that show a range of different phenotypes on different prey and each was independently re-generated in the same background (the parental strain AX4). In all cases, the effect of the mutant matched that expected based on the outcome of the selection screen (Supplementary Data 2 and Supplementary Fig. 5A), with growth performance on each bacteria highly correlated (*r* = 0.89, *p* < 10^−5^) with their relative enrichment or depletion during the screen (Supplementary Fig. 5B).

### Mutations are generally prey-specific and seldom exhibit pleiotropy

Natural *D. discoideum* isolates exhibit frequent swaps in predatory performance across bacterial prey species, with most similarity across the *K. aerogenes*–*K. pneumoniae* pair. This behaviour suggests that most positive pleiotropy should appear between these two species, but that mutations should generally not show positive pleiotropy (unless they are universally deleterious because they disrupt processes required for basic cellular growth or division or affect processes generically associated with growth under lab conditions). To test this idea, we compared the number of mutations that show the same effect (positive or negative) on multiple preys to those that do not. For this, we did not consider mutants with <100 normalised reads in the starting library as they are prone to stochastic dropout (Fig. 3c) (see ‘Methods’ section). This left 902 advantaged or disadvantaged mutants with >100 reads in the start library that can be judged to have increased or decreased in frequency with a high level of confidence. Overall, we find very little positive pleiotropy (164/902 = 18%). This lack of positive pleiotropy is particularly clear for mutants that are advantaged on different bacterial prey, with only 9% of positively pleiotropic mutations (15/164) being advantaged on more than one prey (Fig. 4c) (meaning 91% of positively pleiotropic mutations are disadvantaged on multiple prey). Moreover, of these, only one shows an advantage on three different prey and no mutant had an advantage on all four prey (Fig. 4c). A similar pattern was observed with disadvantaged mutants, although as expected the degree of overlap was higher. Of the mutations that were disadvantaged on more than one prey, 12% (18/149) were disadvantaged on three prey and two (1%) were disadvantaged on all four prey (Fig. 4c). This failure to detect widespread positive pleiotropy is not simply due to experimental error. We were able to estimate the amount of switching in rank through experimental error by splitting mutants into three tranches representing the top 25%, middle 50% and bottom 25%. Next, the number of swaps across each tranche between biological replicates on the same prey was used to estimate the number of expected swaps due to measurement error. In all cases, the number of swaps out of either the top 25% or bottom 25% tranches was higher across prey than expected due to experimental error (Supplementary Tables 1 and 2). This suggests that REMI-seq mutants exhibit relatively little positive pleiotropy across all bacterial prey. Moreover, it is noteworthy that, like the natural isolates, the vast majority (131/164 = 80%) of positive pleiotropy is seen on Gram(−) bacteria (*K. aerogenes* vs. *K. pneumoniae*). Similarly, only 29% (47/164) of the positively pleiotropic mutations show pleiotropy across any pair from different Gram types. Consequently, the pattern of positive pleiotropy mirrors the behaviour observed in wild clones, which suggests that REMI-seq mutations are representative of natural variation.

We next analysed the behaviour of the remaining 738 mutants that do not exhibit positive pleiotropy to determine why there are frequent swaps in predatory behaviour across prey. Frequent swaps can result from extensive but uncorrelated antagonistic pleiotropy (so mutational variation shuffles the pattern of performance across prey in a random fashion), or if mutations are generally conditionally neutral (such that performance on any given prey depends on the overall sum of mutational effects on that prey, which is uncorrelated to the effects of mutations on other prey). To assess this, the 738 mutations were divided into those that show antagonistic pleiotropy (conferring an advantage on at least one bacterium and a disadvantage on at least one other) or conditional neutrality (conferring an advantage or disadvantage on only one prey). This reveals the vast majority of REMI mutations have effects on growth that are prey-specific (Fig. 4d, 698/738 = 95%), the majority of which are disadvantageous (539/698 = 77%). Antagonistic pleiotropy is present but represents only 5% (40/738) of the mutants that do not show positive pleiotropy (Fig. 4e), which corresponds to only ~4% of all mutants (40/902). These results, therefore, suggest that predator variability in nature is likely to be a consequence of the fact that different genes govern predatory success under different conditions, resulting in persistence of mutational variation under relaxed selection, along with a small degree of weak antagonism, potentially reflecting more limited trade-offs.

### Biological processes underlying predation are highly prey-specific

One reason why mutations affecting predation are generally prey-specific could be because distinct physical or biological properties are required to handle different species of bacterial prey. To test this idea, we identified GO terms associated with the genes that, when mutated, result in growth phenotypes (i.e. advantaged or disadvantaged) on each of the four prey types (Supplementary Data 3). We found that enriched GO terms were largely prey-specific. For example, differences between genes that affect growth on the pathogenic *K. pneumoniae* and its congeneric non-pathogenic version *K. aerogenes* suggest that the uptake and killing of *K. pneumoniae* is a crucial step. This is because F-actin rearrangements are required for bacterial uptake, where they drive the formation of the phagocytic cup^46,47^. Consistent with the idea that actin dynamics are crucial for handling this prey, regulators of the actin cytoskeleton such as actin-binding proteins and regulators of GTPase activity^48^ are highly enriched in genes affecting growth on *K. pneumonia*. In addition, the enrichment of metal transporters, especially zinc transporters, is consistent with their involvement in effectively killing bacterial prey through metal poisoning^49^. In contrast, processes associated with growth on *K. aerogenes* appear to be particularly affected by the regulation of cGMP activity, which is required for normal-directed cell motility toward prey^50^. Growth on *K. aerogenes* is also particularly affected by transcriptional regulators. This is consistent with the idea that growth on different bacteria (especially Gram(+) versus Gram(−)) is associated with large scale prey-specific transcriptional changes^51,52^. Indeed, transcriptional changes also appear to be central to growth on *M. luteus*, although in this case, it is the disruption of chromatin remodelling, which is expected to result in global transcriptional changes, that has the biggest effects. Finally, stress response pathways appear to be particularly important for growth on *B. subtilis*. For example, there is an enrichment for genes associated with the ability of cells to deal with free radicals and associated DNA damage, which suggest that this prey exerts particularly toxic effects on *D. discoideum* growth^29,53^. Taken together, these results indicate that large numbers of genes embedded within a diverse set of genetic pathways underlie growth across different bacteria, ranging from the ability to respond to different prey through transcriptional changes, prey uptake, prey killing and resistance to prey mediated toxicity. Since these processes are generally functionally independent, their function is only conditionally required, which can facilitate genetic diversity in heterogeneous environments.

## Discussion

The success of a predator is determined by the efficiency with which it overcomes the various defences of its prey and then converts different prey resources into fitness. In theory, selection to optimise associated traits should result in the evolution of an optimal predator that can outcompete other predators for these resources, which will erode genetic variation. However, we find that co-occurring natural isolates of *D. discoideum* vary widely and unpredictably in the relative efficiency with which they can utilise different prey resources. To understand why we see this pattern, we imposed selection on a pool of insertion mutants by growing them on four diverse prey types. This revealed a large number of genes (902) that impact predator performance across different prey. A relatively small proportion of these genes (164/902 = 18%) exhibit positive pleiotropy that would have transitive effects across prey. Instead, the large majority of REMI mutations (698/902 = 77%) have conditionally neutral, prey-specific effects. Whilst REMI mutants and natural spontaneous mutations could potentially differ in their magnitude of effect, or even the extent of pleiotropy, we believe that the pattern we observe likely captures an important property of genetic architecture. Insertional mutants might logically be expected to result in more extensive pleiotropy because they lead to larger-scale molecular changes, while these effects may be avoided by the subtle changes caused by spontaneous mutations. However, our finding of very limited pleiotropy, with effects often being private to a single growth condition, suggests that our characterisation of pleiotropy has identified a fundamental property of the system. This idea is also supported by data on patterns of gene expression in cells grown on Gram(+) and Gram(−) bacteria, which suggest that different sets of genes are expressed under these conditions^51^. This pattern of gene expression should reduce the opportunity for pleiotropic effects, likely leading to conditional neutrality given that mutations in genes that are not expressed on a given prey should have no effect on growth. Whilst this lack of pleiotropy might imply that natural selection could potentially optimise performance across prey, the lack of consistent selection would allow for the stochastic accumulation of deleterious variation that reduces adaptation^20,25,54^.

While conditional neutrality of mutations is the most common pattern in our mutant screen, we nonetheless see a subset of pleiotropic loci that have a range of patterns of effect. Of the 204 pleiotropic mutations, most show positive pleiotropy (164/204 = 80% of all pleiotropic mutants), meaning that only 20% of pleiotropic mutations show antagonistic effects (which corresponds to only ~4% of all mutations being antagonistically pleiotropic). The majority of the positively pleiotropic mutants (80%) are in the Gram(−) (*K. aerogenes*–*K. pneumoniae)* pair, with only a small fraction (8%) of the antagonistically pleiotropic mutations appears in this pair (which together would make performance across these two more similar than across any other prey combination). This is consistent with observations from the correlations in growth performance in the natural isolates tested (Table 1). Similarly, a very small fraction (9%) shows positive pleiotropy between *M. luteus* and any other species, suggesting that growth on *M. luteus* is distinct from growth across the other bacteria tested. In contrast, the majority of the antagonistically pleiotropic effects (72%) are between *M. luteus* and one of the other bacterial species, further indicating that performance on *M. luteus* is associated with a very different set of characteristics than performance on other bacteria. It is thus reasonable to assume that antagonistic pleiotropy will also have important consequences for shaping variation in predatory performance and preventing the emergence of a single optimal predatory type in nature, when the true diversity of prey species is considered. This constraint is captured by a simple expression, where adaptive evolution of predatory success across all prey will be completely constrained if the average negative correlation across prey is at least as large as −1/(*N* – 1) (where *N* is the number of different prey types), meaning that the average negative correlation need only be very small to prevent adaptive evolution when there is a large number of potential prey (e.g. with just six prey, the average correlation only needs to be −0.2)^55^. Finally, it is important to consider that the impact of these differences on co-existence will be strengthened if there is competition between prey for growth resources. For example, if two predators each have a preferred prey, then whichever prey happens to be more common will lead to the corresponding predator being more common. In turn, this will decrease that prey population and allow the other prey to increase in number, which will promote the fitness of the other predator. This fluctuating red queen dynamic would mean that the predator–prey interaction will generate negative frequency dependence^56^.

Understanding how the diversity and stability of microbial communities is shaped is a major question in biology with wide-ranging implications for ecology, health and disease. Whilst great strides towards understanding genetic variation mediating species interactions have been made through the study of host-parasite systems, there are critical differences that make it difficult to directly translate these findings to predator–prey systems. For example, there can be a very tight relationship between how specific genetic variants in the host and parasite affect each other’s fitness. That sort of interplay can maintain variation through the ‘Fluctuating Red Queen’ processes^57^. In contrast, in predator–prey systems, the fitness of prey is most often tied to generic predator-avoidance traits, like their toxicity or sprint speed, while selection on predators likewise favours generic predatory phenotypes. This can lead to persistent selection at the phenotypic level that drives ongoing evolution through the ‘Escalatory Red Queen process’^57^. Furthermore, because predators typically interact with many preys and vice versa, there is no one-to-one link between their fitness, and selection on each side has to favour general strategies since success is not tied directly to any given ‘opponent’ (i.e. the success of a given genotype of a given bacteria species is not primarily tied to its ability to evade specific genotypes of *D. discoideum*, while nor is the success of any given genotype of *D. discoideum* tied to its ability to consume a given genotype of a given prey species). Consequently, our understanding of the genetics of interspecies interactions outside host-parasite systems remains poor. Such studies are likely to inform how community composition is shaped by phagocytic predation by amoebae to phagocytic immune cells in our bodies. For example, there is evidence to suggest that for some human pathogens, including *Legionella pneumophila*, strong selection to avoid predation by amoebae in their natural habitats has served as ‘training grounds’ for these pathogens that exacerbate their virulence and harmfulness to humans^58^. Understanding the cell, molecular and biochemical properties of the systems underlying predation, as well as the nature of the pleiotropic constraints will provide key insights into how these crucial communities can be maintained or engineered. Our studies reveal that *D. discoideum* is a promising model organism to employ these approaches to better understand interspecies ecological interactions.

## Methods

### Growth and maintenance of strains

We used a set of 24 naturally occurring isolates of *D. discoideum* collected from Little Butts Gap in North Carolina^33^, as well as standard laboratory isolates AX2 or AX4. For growth competition experiments we also generated gene replacement strains in AX2 and AX4 in which the actin 5 gene was replaced by homologous recombination with GFP^59^, to generate AX2-GFP and AX4-GFP. Cells were grown and maintained on SM plates (Formedium) spread evenly with a single species of bacteria as a food source (see below). The bacterial strains used were *K. pneumoniae* 52145 isogenic mutant^60^, the isogenic *Pseudomonas aeruginosa* strain PT531 (rhlR-lasR non-virulent mutant^61^), *Escherichia coli* B/r^62^, nonsporulating *Bacillus subtilis* 36.1^63^, and *Micrococcus luteus*^64^ all kindly provided by Jason King (University of Sheffield). The axenic strains AX2 and AX4 as well as all mutants generated in these strains were grown and maintained at 22 °C either on SM plates, or in HL-5 media containing glucose (Formedium) and 1× PVS on 10 cm tissue culture plates. 100× PVS comprises 3 g penicillinG, 5 g streptomycin sulphate, 10 mg folic acid, 30 mg vitamin B12 in 500 ml H_2_O, filter sterilised, and stored at 4 °C in the dark.

### Growth assays and analysis of growth traits

For growth competitions, cells of both AX2-GFP and competitor natural genotypes were initially grown clonally on SM plates containing the appropriate food source. For Gram(−) competitions, all strains were grown up on *K. aerogenes*. For Gram(+) competitions, strains were grown up on the species of bacteria to be competed on to both to allow for sufficient time for *D. discoideum* to adapt to growth on Gram(+) bacterial food^51^, as well as to prevent any crossover *K. aerogenes* contamination onto the competition plate. 2.5–5 × 10^5^ spores were mixed with 400 µl of an overnight culture of the appropriate bacterial food and incubated at 22 °C for ~36 h or until the amoebae had eaten most of the bacteria but had not yet begun to aggregate. Bacteria and amoebae were harvested, and amoebae were washed in KK2 buffer (16.1 mM KH_2_PO_4_, 3.7 mM K_2_HPO_4_) repeatedly until most of the bacteria had been removed. Cells from each genotype were resuspended to 10^7^ cells/ml and mixed 1:1 with AX2-GFP. The precise starting frequency of each strain (relative proportion of GFP-labelled to unlabelled cells) was determined before the start of the competition by flow cytometry (Beckmann Coulter CyAn ADP, running Summit software). To start the competition, 2 × 10^4^–1 × 10^6^ amoebae (depending on the bacterial prey) were mixed with 400 µl of an overnight culture of bacteria, spread evenly on an SM plate, and left to grow for 48 h in competition until most of the bacteria had been eaten. Cells were harvested and washed in KK2, and the relative proportion of GFP-labelled to unlabelled cells was determined by flow cytometry to get a measure of the change in frequency at the end of the competition. We used the difference in the proportional representation of a strain in competition with AX2-GFP (end frequency minus start frequency) as the measure of the relative growth rate of each strain on each bacterium. The strain NC60.1 failed to grow at all on *B. subtilis* in any of the replicates. To capture this low performance of NC60.1, we assigned the theoretically maximum score of −0.5, which allowed us to include NC60.1 in the analysis of relative predatory performance across bacterial species (giving it the lowest possible performance on *B. subtilis*, but otherwise not affecting the estimation of relative growth rates).

We calculated the means and standard errors of growth of each strain on each bacteria as the expected values from a mixed model (fitted using the lmer function in the lme4 package for R^65^) with strain, bacteria and strain-by-bacteria as fixed effects and block and bacteria-by-block as random effects (where the strain-by-bacteria expected values provide the mean estimates). We then used these estimates to compare the means of the strains on each bacterium by constructing two-sample *t*-tests, with significance determined using an overall false discovery rate of 5% based on the Benjamini–Hochberg procedure^66^. We then determined the number of significant ‘wins’ and ‘losses’ each strain had against each of the other seven strains (in terms of significant differences in growth) and used their win-loss record (i.e. the difference between the number of wins and losses) to rank strains on each bacteria (1 = best performing strain, 8 = worst). We also used the head-to-head comparison of strains on each bacterium to identify specific strain pairs that showed significant swaps in their relative growth rate in which each strain in the given pair shows both a significantly higher and significantly lower growth than the other strain across different bacteria.

### Co-existence experiments

Growth competitions were performed exactly as described above except those overnight preparations of bacteria were first mixed together (*K. aerogenes*/*K. pneumoniae* or *E. coli*/*K. pneumoniae*) at different ratios (1:99, 5:95, 10:90, 25:75, 40:60, 50:50, 60:40, 75:25, 90:10, 95:5, 99:1), and all *D. discoideum* cells were initially grown up on clearing plates with *K. aerogenes* as the single prey source. Growth competitions were performed using AX2-GFP against the natural isolates NC80.1 and NC85.2. These strains were chosen because they were found to have switches in competitive performance on different prey: AX2-GFP performed better than NC80.1 and NC85.2 on *K. pneumoniae* but performed worse than both strains on either *K. aerogenes* or *E. coli* (Fig. 1b).

### Spike-in experiment to test sensitivity and quantification of mutant detection

Cells from 32 REMI mutants with defined insertion sites were grown separately on SM plates in association with *K. aerogenes*. After washing in KK2 buffer, cells of each genotype were resuspended to 10^7^ cells/ml and mixed at varying frequencies to generate four pools of cells (Supplementary Data 1) where the relative frequency of ‘1000’ was 10^8^ cells, ‘100’ = 10^7^ cells, ‘10’ = 10^6^ cells and ‘1’ = 10^5^ cells.

### Preparation of target for Illumina sequencing and analysis of reads

Protocols described at REMI-seq.org were followed. Briefly, nuclei were isolated from cells grown in association with *K. aerogenes* on SM agar plates Genomic DNA was extracted and digested with *Mme*I and *I-Sce*I to excise the genomic DNA – insert DNA junction. Indexed adapters (D7 and D5) were ligated to the digested DNA, before amplification by PCR using primers specific to the D7 and D5 adapters. Size selection by electrophoresis using a 3.5% low melting temperature agarose gel (NuSieve™ GTG™ Agarose, Lonza) was used to enrich the DNA of interest. The ~183 bp band was excised and size selected a second time before sequencing using an Illumina MiSeq with a MiSeq Reagent Kit v3 (75 cycles) or NextSeq® 500 Sequencer with a High Output Kit v2 (75 cycles). Adapter sequences were trimmed before each read was checked for the presence of the vector sequence using an R Shiny app (https://github.com/NicoleGruenheit/REMI-seq-screen). The script extracts 19/20 bp tag sequence as well as vector-specific indices. Tag and index combinations were counted and compared to a pre-computed lookup table to determine the total number of mappable reads, number of unique tags, number of tags in the inverted repeat (Chromosome DDB0232429, 2263132 to 3015703 is repeated between bases 3016083 and 3768654), and number of non-unique tags. Raw read counts were normalised to the total number of reads per sample, in order to calculate the total number of reads per insertion point (for upstream and downstream tag). Finally, insertion points were filtered for PCR artefacts (low occurrence of the different index), and non-unique tags.

### Parallel genetic selections for growth on different Gram(–) and Gram(+) bacteria

The mutant library was initially thawed directly into filter sterilised HL-5 media + 1×PVS and allowed to recover for 36 h. Cells were then collected and washed twice in KK2 before being resuspended at 10^7^ cells/ml. 25 μl of the suspension (2.5 × 10^5^ cells) was then mixed with 400 μl of an overnight culture of each of the four bacteria and plated in duplicate for each selection onto an SM plate. For each biological replicate of each screen, two plates were prepared in this way to prevent bottlenecking of the mutant library during serial transfer. After 48 h (approximately 10 generations of growth), when the cells had eaten most of the bacteria but had not yet begun to aggregate and enter development, cells from both plates were pooled, harvested and washed by repeated centrifugation at 500 g in KK2 until all the bacteria had been removed. Cells were then resuspended at 1 × 10^7^ cells/ml, and 2.5 × 10^5^ cells were replated serially on fresh bacteria to begin another ‘round’ of selection. An aliquot of cells was also resuspended at 5 × 10^7^ cells/ml in freezing media (50% FBS, 42.5% HL-5, 7.5% DMSO) and stored at −80 °C. The screens were continued for ~100 generations (10–13 rounds) on each bacterium except *K. aerogenes*, which was continued for 200 generations (20 rounds).

### Analysis of mutant pools before and after selection

The mutant library was prepared for Illumina sequencing in triplicate as described above. Sequencing of the start library (Fig. 4a) identified unique insertions at a total of 12,479 different loci. These mutations occurred in the coding sequence of 5050 different genes (~38% of all genes in the genome), or 5680 genes (~42%) when promoter insertions (within 500 bp of a single start codon) were included. To determine possible bias in the representation of mutants in the starting pool, for example as a consequence of not hitting essential genes, we performed GO analysis on the starting library of 5680 genes. Consistent with this idea, GO terms associated with core cellular machineries, including those involved in translation and endocytosis, are underrepresented (Supplementary Data 3), while mutants present in the library are associated with these GO terms generally have lower than average frequencies (Supplementary Data 2).

After sequencing of all eight pools at the end of the selections (Supplementary Fig. 3), phenotypes were assigned to mutants under each condition, To do this, mutants were first binned according to their mean normalised starting read counts (bin 100 = <100 reads, bin 1000 = 100–1000 reads, bin 10,000 = >1000 reads) and log-fold change values relative to starting read count were calculated for each insertion mutant in each bin. To allow for comparisons of mutants across time, we normalised these data to have a mean of 0 and a standard deviation of 1 for each of the three bins of mutants (Supplementary Data 2). Mutants with a mean *z*-score >1.5 (i.e. >1.5 standard deviations from the mean of that bin) were considered to have an advantage under that condition as long as each replicate had an end read count >100, and mutants with a *z*-score < −1 a disadvantage (Supplementary Fig. 4). Mutants with fewer than 100 starting read counts that dropped out were discounted from this analysis because the technical dropout rate for these mutants was very high (Fig. 3b). Hierarchically clustered *z-*score data across time for each of the screens were visualised using the ggplot2 package in R^67^, Venn diagrams were generated using the online tool Venny^68^.

### Growth mutant validation

A selection of four mutants showing varying growth phenotypes on different bacteria (Supplementary Data 2, Supplementary Fig. 5) was validated by growth competition assays with GFP-labelled AX4. To perform the growth competitions, cells of both AX4-GFP and competitor mutants were initially grown in parallel in tissue culture. Cells were then harvested, washed twice in KK2 and resuspended to 1 × 10^7^ cells/ml. To start each competition, mutant clones as well as the parental AX4 control were mixed 1:1 with AX4-GFP, and 2.5 × 10^5^ cells were then plated on either a single SM plate in association with 400 μl bacteria in duplicate for two technical replicates per competition. Cells were allowed to grow together for 48 h (10 generations) and were then washed and replated on an overnight culture of bacteria onto a fresh plate three times for a total of ~30 generations of growth in competition (three rounds). The relative proportion of GFP-labelled to unlabelled cells was scored at the start as well as the endpoint of the competitions by flow cytometry (Attune NxT Flow cytometer). Competition data was normalised to wild-type labelled vs wild-type unlabelled controls. Data are expressed as a change in frequency (end frequency minus start frequency) from at least two independent biological replicates.

### GO analysis

To perform GO analyses on the mutant lists, we used the GSEAbase R package^69^ using a cut-off of *p* < 0.05 for significantly over- or underrepresented GO terms. Gene lists for genes with phenotypes on each bacteria was compared against a universe of every gene represented in the starting library (Supplementary Data 2).

### Reporting summary

Further information on research design is available in the Nature Research Reporting Summary linked to this article.

## Supplementary information

Supplementary Information

Reporting Summary

Description of Additional Supplementary Files

Supplementary_Data 1

Supplementary_Data_2

Supplementary_Data_3

## Data Availability

All data generated in this study that are necessary to interpret, verify and extend the research in the article are provided in the Supplementary Information and Source Data files provided with this paper. Raw reads of all samples were uploaded to the NCBI SRA database (accession numbers PRJNA524784, PRJNA524539 and PRJNA785534). Source data are provided with this paper.

## Source data

Source data

## Code availability

The R Shiny apps to analyse pools of REMI-seq mutants or grids can be found here https://github.com/NicoleGruenheit/REMI-seq-screen^70^ and https://github.com/NicoleGruenheit/grid_analyser^71^. Detailed protocols, computational pipelines and further information can also be found at www.remi-seq.org.
