# Supplementary material for: The genetic architecture underlying prey-dependent performance in a microbial predator
Source: Nat Commun. 2022 Jan 14;13:319. doi: 10.1038/s41467-021-27844-x (PMC8760311; doi:10.1038/s41467-021-27844-x)
Supplement: Supplementary file 1 — Supplementary Information [file 41467_2021_27844_MOESM1_ESM.pdf]

Supplementary Figure 1

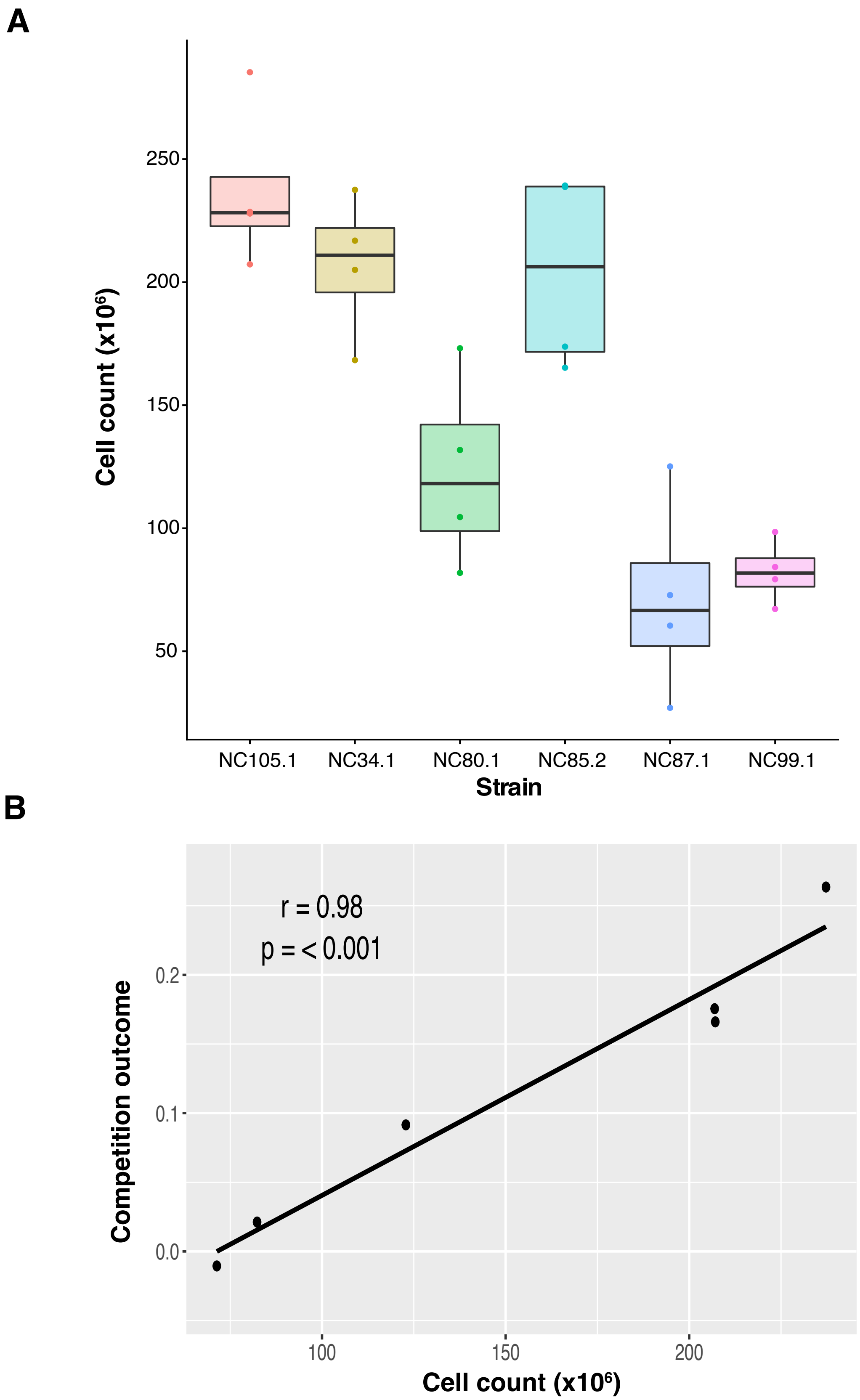

Supplementary Figure 1: Variation in growth fitness reflects differences in resource utilisation rather than interference competition. Representative strains from across the K. aerogenes growth hierarchy were grown clonally with K. aerogenes. Four biological replicates were performed per strain.
A. Box plots of the total cell numbers of each strain after 48 hours of growth. The individual points represent the means of the technical replicates for each of the four biological replicates. The thick horizontal line gives the median, with the box spanning from the 25th to 75th percentiles. The limits of the vertical 'whisk-ers' indicate the maximum and minimum observations, except where an observation is free-floating, which indicates an outlier. Source data are provided in Supplementary file 1.
B. Total cell numbers from clonal growth plates are highly correlated with the outcome in the competition plates. The Pearson correlation is shown ($p = 0.000596$). Source data are provided as a Source Data file.

Supplementary Figure 2

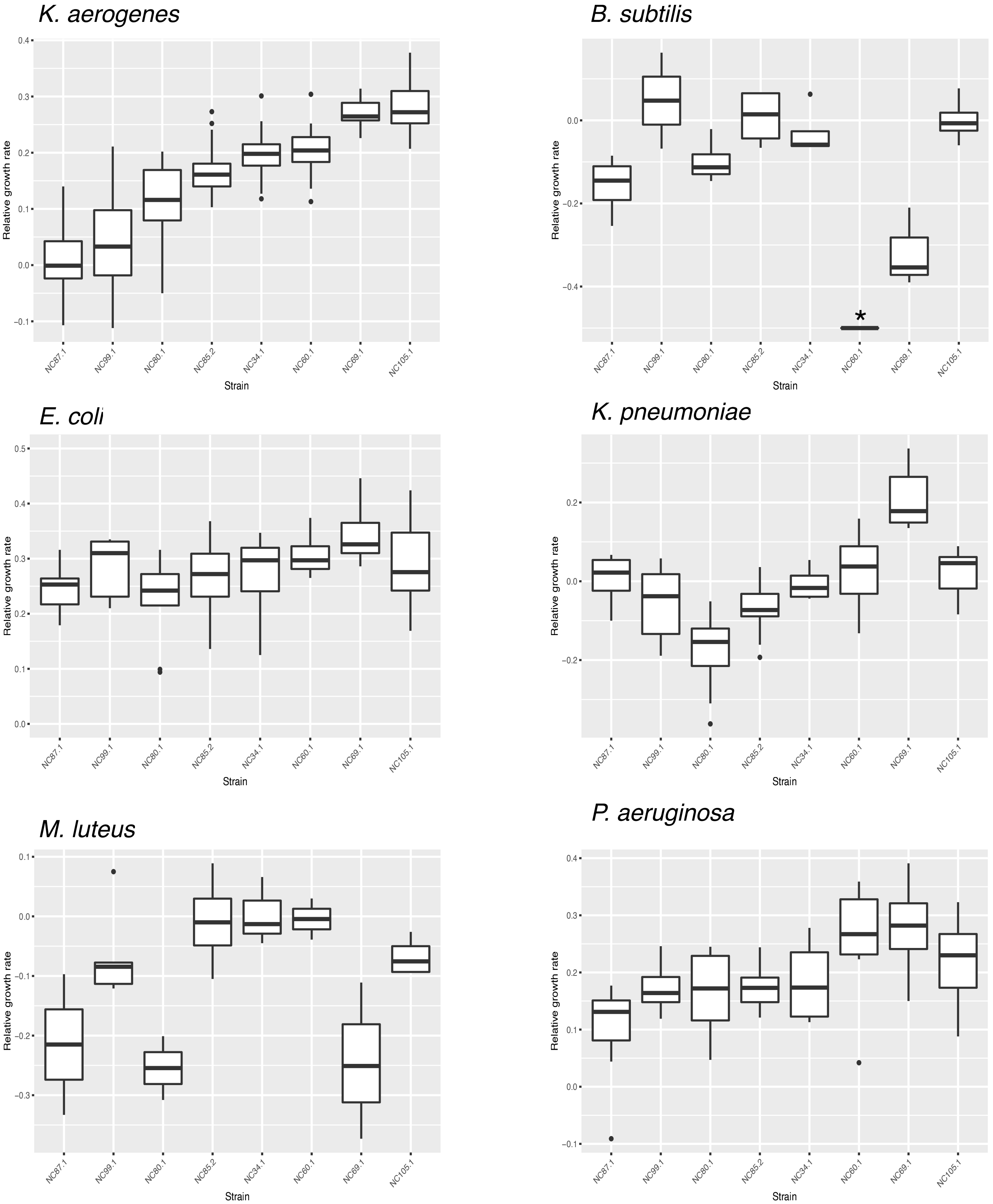

Supplementary Figure 2: Relative growth rates of a subset of D. discoideum strains on different bacterial prey. Growth competitions were performed using eight strains from the top, middle and bottom of the K. aerogenes growth hierarchy against the AX2-GFP. Competitions were performed on 6 different species of bacteria (K. aerogenes (Ka), B. subtilis (Bs), E. coli (Ec), K. pnuemoniae (Kp), M. luteus (Ml), and P. aeruginosa (Pa)). Horizontal lines within the boxes indicate median values, the boxes the inter-quartile range, whiskers extend to the largest and smallest values within 1.5 times of the 75th percentile and 25th percentile, respectively, and dots represent outliers outside these ranges. For each plot, strains are ordered by their relative growth rate on K. aerogenes. NC60.1 was not detectable at the end of the growth competition on B. subtilis (marked with an asterisk). Growth rates are normalised to the AX2-GFP control (relative growth of control = 0) and were performed at least twice independently. Source data are provided as a Source Data file.

Supplementary Figure 3

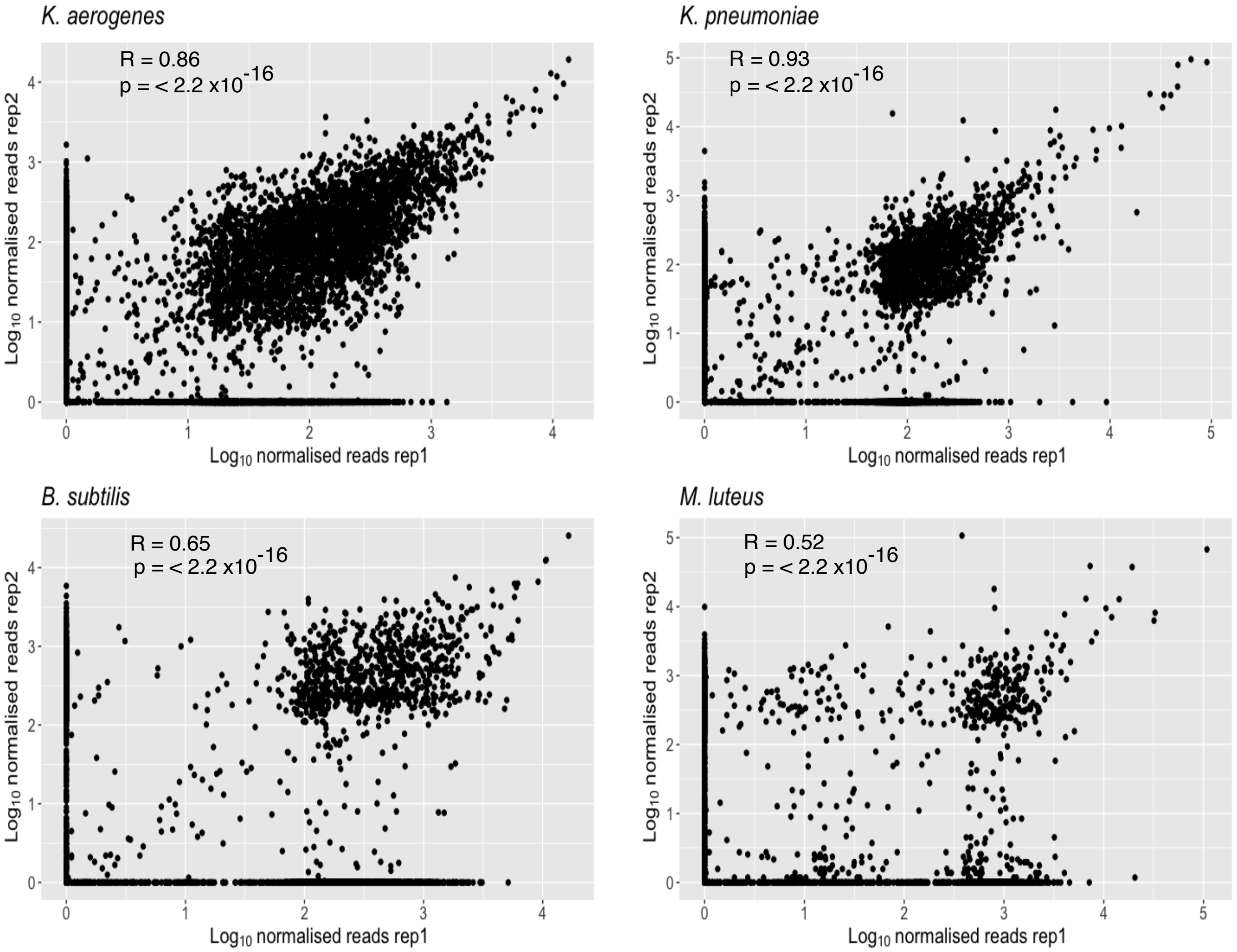

Supplementary Figure 3: Read counts for each mutant are highly correlated between biological replicates. The Pearson correlation is shown (p = 10-16)

Supplementary Figure 4

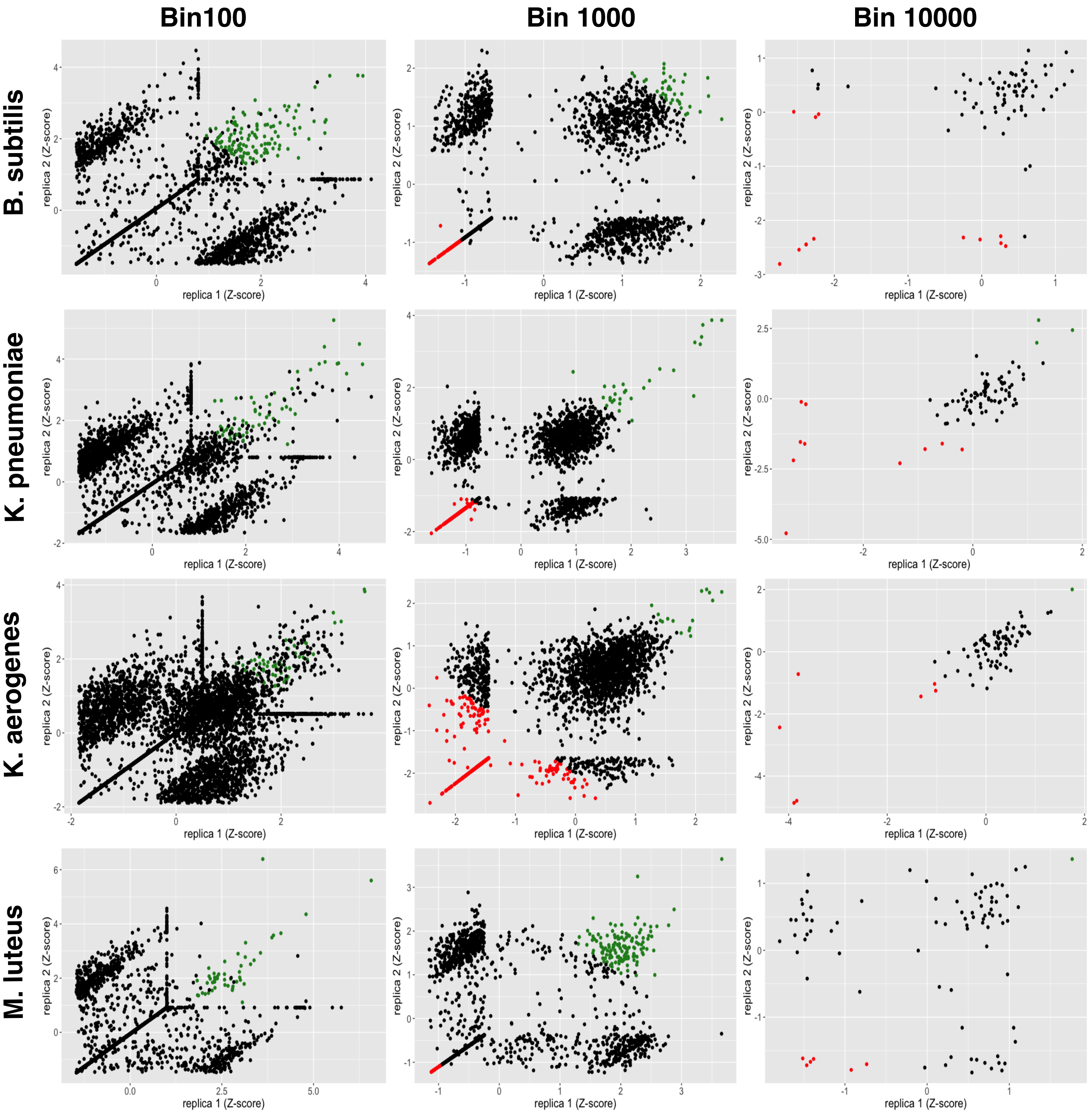

Supplementary Figure 4: Identification of mutants with growth advantages and disadvantages. The abundance (read count) of each mutant after selection was compared to the start pool. Mutants were first divided into bins based on their read count in the start pool in order to identify mutants that deviated in abundance (z-score) significantly from other mutants with similar read counts. Mutants with a mean z-score of >1.5 were considered to have increased in abundance if their end normalized read counts were also >100 in both replicas (green). Mutants with a mean z-score of < −1 were considered to have decreased. In bin <100, the variation due to technical dropouts resulted in a high false discovery rate, and mutants that decreased were not considered. Replicas are highly correlated.

Supplementary Figure 5

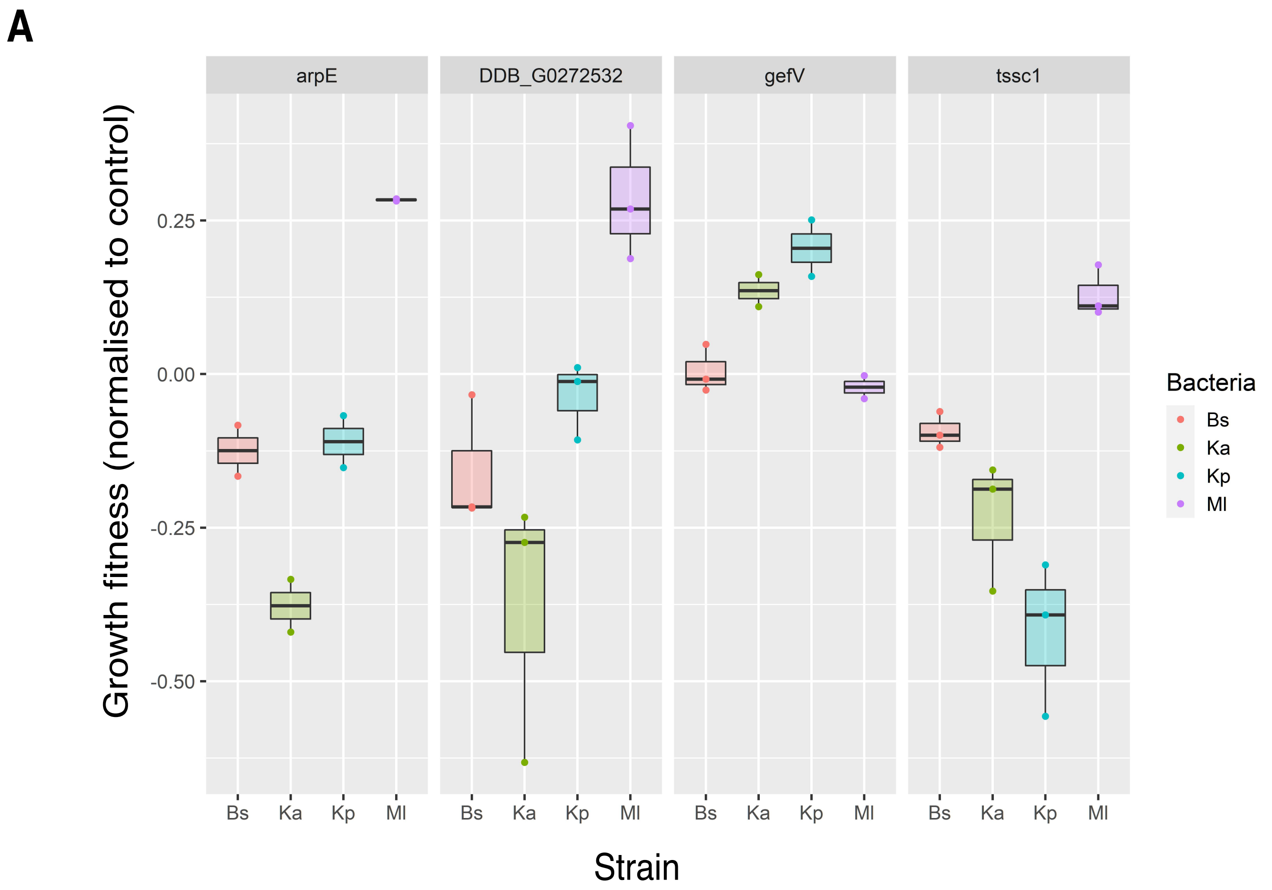

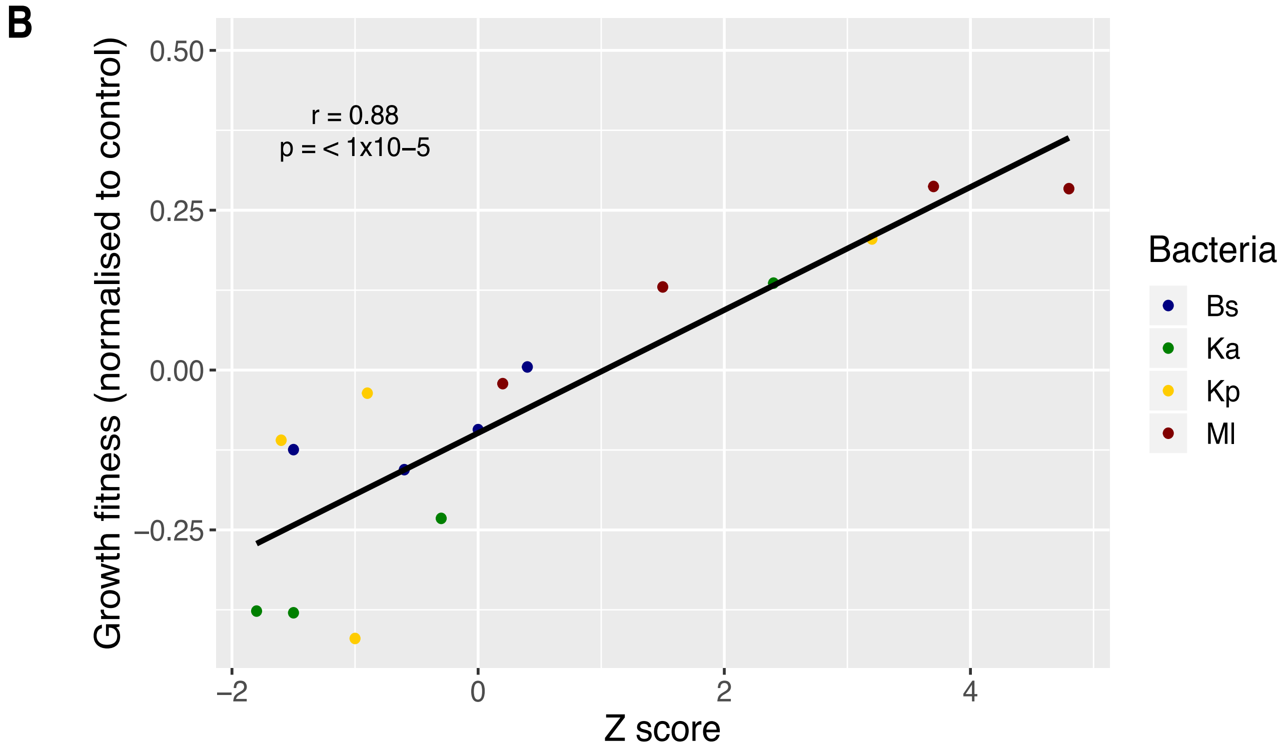

Supplementary Figure 5: Validation of mutant identification by REMI-seq. A. Boxplots of the relative growth rates of four mutants identified by REMI-seq. Independent isolates of four different mutants showing a variety of different phenotypes identified by REMI-seq were tested for growth effects in competition with the parental AX4 strain on four different bacterial species. The bacterial species are identified using the first letter of their genus and species (see the legend for Supplementary Fig. 2). Sample sizes are indicated by the number of data points in each individual boxplot. The thick horizontal line gives the median, with the box spanning from the 25th to 75th percentiles. The limits of the vertical 'whiskers' indicate the maximum and minimum observations, except where an observation is free-floating, which indicates an outlier. Source data are provided as a Source Data file. B. REMI-seq can quantitatively predict fitness effects in growth competition. There is a strong correlation between the experimentally measured growth rate of each mutant and the relative change in abundance of each mutant inferred by REMI-seq at the final round of selection on each bacterium (z-score). The Pearson correlation is shown.

Supplementary Table 1: Comparison of the degree of positive pleiotropy across bacteria

	K. aerogenes	K. pneumonia	B. subtilis	M. luteus
K. aerogenes		0.39	0.2	-0.02
K. pneumonia	0.61		0.61	0.28
B. subtilis	0.24	0.46		0.69
M. luteus	-0.02	0.2	0.59	

Supplementary Table 1: Comparison of the degree of positive pleiotropy across bacteria. Mutants with a read count >100 in the starting library were ranked from top to bottom on each bacteria after selection. Mutants in the top or bottom 25% were compared across different bacteria. The degree of overlap was converted into a 'pleiotropy index' which accounts for the fraction of overlap observed between biological replicates (reproducibility score) as this would be expected to represent perfect positive pleiotropy (index =1). It also accounts for the lowest expectation in which mutant ranking is random (random expectation) which equates to the tranche size (index = 0). Pleiotropy index = (observed – random expectation)/(reproducibility score − random expectation). Comparisons show values when reproducibility scores are used from each pairing in turn (i.e., Ka v Kp when reproducibility scores from Ka (rows) or Kp (columns) are used.

Supplementary Table 2: Probabilities that REMI-seq mutants exhibit complete positive pleiotropy or no pleiotropy on different bacteria based binomial expectations.

Comparison	Bacteria (replicate)	Positive pleiotropy		Random		Log likelihood ratio (pleiotropy/random)	
		**By bact.**	**Total**	**By bact.**	**Total**	**By bact.**	**Total**
Ka / *Kp*	*Ka*	45.4	44.9	23.8	46.2	-22.2	-1.5
	Kp	8.4		23.7		14.0	
Ka / *Bs*	*Ka*	78.7	126.7	6.7	12.4	-72.6	-116.6
	Bs	51.0		6.7		-45.8	
Ka / *Ml*	*Ka*	127.6	226.7	0.1	0.3	-128.9	-232.0
	Kp	101.5		0.8		-104.6	
Kp / *Bs*	*Ka*	8.4	30.3	23.4	45.6	13.4	11.9
	Bs	25.6		23.5		-3.4	
Kp / *Ml*	*Ka*	27.1	84.6	5.3	10.0	-22.3	-77.0
	Kp	62.7		5.5		-58.6	
Bs / *Ml*	*Ka*	12.4	27.7	42.2	83.0	28.6	52.9
	Kp	16.6		42.2		24.4	

Supplementary Table 2: Probabilities that REMI-seq mutants exhibit complete positive pleiotropy or no pleiotropy on different bacteria based binomial expectations. For each pair of bacteria, the two rows give the probability that the two replicates on the listed bacteria could be replicates on the other bacteria (positive pleiotropy, by bacteria) or that the overlap is due to random change (random, by bacteria). The 'total' columns represent the combined probability of the two tests. The log likelihood ratio is based on the probability of the observed degree of overlap between replicates on the two bacteria assuming perfect positive pleiotropy compared to the random expectation. Probabilities of the replicates by were combined using the Z-transform test (Whitlock, M. C. Combining probability from independent tests: the weighted Z-method is superior to Fisher's approach. J Evol Biol 18, 1368-1373 (2005)). All values represent −log10[p].
