# Supplementary material for: The genetic architecture underlying prey-dependent performance in a microbial predator
Source: Nat Commun. 2022 Jan 14;13:319. doi: 10.1038/s41467-021-27844-x (PMC8760311; doi:10.1038/s41467-021-27844-x)
Supplement: Supplementary file 2 — Reporting Summary [file 41467_2021_27844_MOESM2_ESM.pdf]

nature research

Corresponding author(s): Christopher Thompson, Jason Wolf

Last updated by author(s): 2021-27-03

Reporting Summary

Nature Research wishes to improve the reproducibility of the work that we publish. This form provides structure for consistency and transparency in reporting. For further information on Nature Research policies, see our Editorial Policies and the Editorial Policy Checklist.

Statistics

For all statistical analyses, confirm that the following items are present in the figure legend, table legend, main text, or Methods section.

n/a	Confirmed	
☐	☒	The exact sample size (n) for each experimental group/condition, given as a discrete number and unit of measurement
☐	☒	A statement on whether measurements were taken from distinct samples or whether the same sample was measured repeatedly
☐	☒	The statistical test(s) used AND whether they are one- or two-sided<br>*Only common tests should be described solely by name; describe more complex techniques in the Methods section.*
☐	☒	A description of all covariates tested
☐	☒	A description of any assumptions or corrections, such as tests of normality and adjustment for multiple comparisons
☐	☒	A full description of the statistical parameters including central tendency (e.g. means) or other basic estimates (e.g. regression coefficient) AND variation (e.g. standard deviation) or associated estimates of uncertainty (e.g. confidence intervals)
☐	☒	For null hypothesis testing, the test statistic (e.g. F, t, r) with confidence intervals, effect sizes, degrees of freedom and P value noted<br>*Give P values as exact values whenever suitable.*
☒	☐	For Bayesian analysis, information on the choice of priors and Markov chain Monte Carlo settings
☒	☐	For hierarchical and complex designs, identification of the appropriate level for tests and full reporting of outcomes
☐	☒	Estimates of effect sizes (e.g. Cohen's d, Pearson's r), indicating how they were calculated

Our web collection on statistics for biologists contains articles on many of the points above.

Software and code

Policy information about availability of computer code

Data collection	FACs analyis: Beckmann Coulter CyAn ADP, running Summit software
Data analysis	mixed model fitted using the lmer function in the lme4 package for R<br>Sequences were checked for the presence of the vector sequence [GC]AT[CG]CGTTGGA using an R Shiny app (https://github.com/NicoleGruenheit/REMI-seqscreen)<br>Hierarchically clustered z-score data across time for each of the screens were visualised using the ggplot2 package in R 59, venn diagrams were generated using the online tool Venny<br>To perform GO analyses on the mutant lists, we used the GSEAbase R package

For manuscripts utilizing custom algorithms or software that are central to the research but not yet described in published literature, software must be made available to editors and reviewers. We strongly encourage code deposition in a community repository (e.g. GitHub). See the Nature Research guidelines for submitting code & software for further information.

Data

Policy information about availability of data

All manuscripts must include a data availability statement. This statement should provide the following information, where applicable:

- Accession codes, unique identifiers, or web links for publicly available datasets
- A list of figures that have associated raw data
- A description of any restrictions on data availability

All raw data has been submitted as supplementary files. Accession codes, unique identifiers, or web links have been provided for publicly available datasets

Field-specific reporting

Please select the one below that is the best fit for your research. If you are not sure, read the appropriate sections before making your selection.

☒ Life sciences ☐ Behavioural & social sciences ☐ Ecological, evolutionary & environmental sciences

For a reference copy of the document with all sections, see nature.com/documents/nr-reporting-summary-flat.pdf

Life sciences study design

All studies must disclose on these points even when the disclosure is negative.

Sample size	Data was generated for all wild strains that were available from the Dicty Stock Center to provide the largest possible dataset for analyses. Representative Dictyostelium or bacterial strains were chosen for detailed analyses as described in the manuscript. For bacterial growth assays 9 experiments were performed with at least 3 replicates of each combination. For REMI-seq experiments, the number of replicated was limited to two per competition due to constraints in sequencing depth
Data exclusions	No data were excluded from analyses
Replication	To validate REMI-seq mutants, experiments were replicated with individual clones that had been isolated independently
Randomization	All experimental components were fully combinatorial
Blinding	All plates/samples were coded to ensure that the experimentor was blind to samples.

Reporting for specific materials, systems and methods

We require information from authors about some types of materials, experimental systems and methods used in many studies. Here, indicate whether each material, system or method listed is relevant to your study. If you are not sure if a list item applies to your research, read the appropriate section before selecting a response.

Materials & experimental systems

n/a	Involved in the study	
☒	☐	Antibodies
☒	☐	Eukaryotic cell lines
☒	☐	Palaeontology and archaeology
☐	☒	Animals and other organisms
☒	☐	Human research participants
☒	☐	Clinical data
☒	☐	Dual use research of concern

Methods

n/a	Involved in the study	
☒	☐	ChIP-seq
☐	☒	Flow cytometry
☒	☐	MRI-based neuroimaging

Animals and other organisms

Policy information about studies involving animals; ARRIVE guidelines recommended for reporting animal research

Laboratory animals	No animals used in the study
Wild animals	the study did not involve wild animals
Field-collected samples	We used a set of 24 naturally occurring isolates of D. discoideum collected from Little Butts Gap in North Carolina 32, as well as standard laboratory isolates AX2 or AX4. For growth competition experiments we also generated gene replacement strains in AX2 and AX4 in which the actin 5 gene was replaced by homologous recombination with GFP 50, to generate

AX2-GFP and AX4-GFP. Cells were grown and maintained on SM plates (Formedium) spread evenly with a single species of bacteria as a food source

Ethics oversight	no ethical approval required for D. discoideum

Note that full information on the approval of the study protocol must also be provided in the manuscript.

Flow Cytometry

Plots

Confirm that:

☐ The axis labels state the marker and fluorochrome used (e.g. CD4-FITC).

☐ The axis scales are clearly visible. Include numbers along axes only for bottom left plot of group (a 'group' is an analysis of identical markers).

☐ All plots are contour plots with outliers or pseudocolor plots.

☒ A numerical value for number of cells or percentage (with statistics) is provided.

Methodology

Sample preparation	Bacteria and amoebae were harvested, and amoebae washed in KK2 buffer (16.1mM KH2PO4, 3.7mM K2HPO4) repeatedly until most of the bacteria had been removed. Cells from each genotype were resuspended to 107 cells/ml and mixed 1:1 with AX2-GFP. The precise starting frequency of each strain (relative proportion of GFP-labeled to unlabeled cells) was determined before the start of the competition by flow cytometry (Beckmann Coulter CyAn ADP, running Summit software). To start the competition, 2x104 – 1x106 amoebae (depending on the bacterial prey) were mixed with 400μl of an overnight culture of bacteria, spread evenly on an SM plate, and left to grow for 48 hours in competition until most of the bacteria had been eaten. Cells were harvested and washed in KK2, and the relative proportion of GFP-labeled to unlabeled cells determined by flow cytometry to get a measure of the change in frequency at the end of the competition. We used the difference in the proportional representation of a strain in competition with AX2-GFP (end frequency minus start frequency) as the measure of relative growth rate of each strain on each bacterium.
Instrument	Beckmann Coulter CyAn ADP
Software	Summit
Cell population abundance	Analysis was performed to count relative proportions, not to purify populations
Gating strategy	Gating strategy was determined by running wild type, unlabelled cells through the sorter in order to define gating that allows no spill over

☐ Tick this box to confirm that a figure exemplifying the gating strategy is provided in the Supplementary Information.
