# Supplementary material for: The genetic architecture underlying prey-dependent performance in a microbial predator
Source: Nat Commun. 2022 Jan 14;13:319. doi: 10.1038/s41467-021-27844-x (PMC8760311; doi:10.1038/s41467-021-27844-x)
Supplement: Supplementary file 3 — Description of Additional Supplementary Files [file 41467_2021_27844_MOESM3_ESM.pdf]

Title: Supplementary Data 1: Dropout rate of mutants compared to read count and table of insertion mutants used in spike-in study to test the quantitative range of REMI-seq.

Description: Dropout rates of mutants in different read count bins (**Sheet 1**). 32 mutants with known insertion points were divided into four groups (A – D; **Sheet 2**). Mutants were then mixed according to the experimental design table (**Sheet 3**), where mutants were added to four different pools at varying quantities. Tags were then prepared using the REMI-seq protocol and sequenced on a MiSeq. Counts for each mutant (**Sheet 4**) were normalised according to the total number of reads per sample.

Title: Supplementary Data 2: Read count data before and after selections for growth on different bacteria.

Description Normalised read count data is shown for each detectable insertion mutant for each replicate of each condition, as well as *z*-score values.

Title: Supplementary Data 3: GO term analysis of starting library genes and genes associated with growth phenotypes on different bacteria. I

Description Individual sheets list GO terms associated with each bacterial species up to p-value <= 0.1. GO terms are shown in individual sheets for all mutants, advantaged or disadvantaged mutants.
